# A Simple Prognostic Score for Critical COVID-19 Derived from Patients without Comorbidities Performs Well in Unselected Patients

**DOI:** 10.3390/jcm11071810

**Published:** 2022-03-25

**Authors:** Vasiliki E. Georgakopoulou, Nikolaos I. Vlachogiannis, Dimitrios Basoulis, Irene Eliadi, Georgios Georgiopoulos, Georgios Karamanakos, Sotiria Makrodimitri, Stamatia Samara, Maria Triantafyllou, Pantazis M. Voutsinas, Fotinie Ntziora, Mina Psichogiou, Michael Samarkos, Petros P. Sfikakis, Nikolaos V. Sipsas

**Affiliations:** 1 Infectious Diseases and COVID-19 Unit, General Hospital of Athens Laiko, Medical School, National and Kapodistrian University of Athens, 11527 Athens, Greece; vaso_georgakopoulou@hotmail.com (V.E.G.); dimitris.bassoulis@gmail.com (D.B.); eirini.iliadi@gmail.com (I.E.); gkaramanakos@gmail.com (G.K.); sotiriamakrod@yahoo.gr (S.M.); stsamara@yahoo.gr (S.S.); mariatriantafyllou88@gmail.com (M.T.); pantazis88@gmail.com (P.M.V.); mpsichog@med.uoa.gr (M.P.); msamarkos@med.uoa.gr (M.S.); 2 First Department of Propaedeutic Internal Medicine and Joint Academic Rheumatology Program, Medical School, National and Kapodistrian University of Athens, 11527 Athens, Greece; nvlachog@med.uoa.gr (N.I.V.); ntziora@laiko.gr (F.N.); psfikakis@med.uoa.gr (P.P.S.); 3 Department of Clinical Therapeutics, Medical School, National and Kapodistrian University of Athens, 11528 Athens, Greece; georgiopoulosgeorgios@gmail.com; 4 Pathophysiology Department, Medical School, National and Kapodistrian University of Athens, 11527 Athens, Greece

**Keywords:** COVID-19, comorbidities, prognosis, vaccination, critical disease

## Abstract

We aimed to search for laboratory predictors of critical COVID-19 in consecutive adults admitted in an academic center between 16 September 2020–20 December 2021. Patients were uniformly treated with low-molecular-weight heparin, and dexamethasone plus remdesivir when SpO2 < 94%. Among consecutive unvaccinated patients without underlying medical conditions (*n* = 241, 49 year-old median, 71% males), 22 (9.1%) developed critical disease and 2 died (0.8%). White-blood-cell counts, neutrophils, neutrophil-to-lymphocyte ratio, CRP, fibrinogen, ferritin, LDH and γ-GT at admission were each univariably associated with critical disease. ROC-defined cutoffs revealed that CRP > 61.8 mg/L, fibrinogen > 616.5 mg/dL and LDH > 380.5 U/L were each associated with critical disease development, independently of age, sex and days from symptom-onset. A score combining higher-than-cutoff CRP (0/2), LDH (0/1) and fibrinogen (0/1) predicted critical disease (AUC: 0.873, 95% CI: 0.820–0.926). This score performed well in an unselected patient cohort (*n* = 1228, 100% unvaccinated) predominantly infected by the alpha variant (AUC: 0.718, 95% CI: 0.683–0.753), as well as in a mixed cohort (*n* = 527, 65% unvaccinated) predominantly infected by the delta variant (AUC: 0.708, 95% CI: 0.656–0.760). Therefore, we propose that a combination of standard biomarkers of acute inflammatory response, cell death and hypercoagulability reflects the severity of COVID-19 per se independently of comorbidities, age and sex, being of value for risk stratification in unselected patients.

## 1. Introduction

Severe acute respiratory syndrome coronavirus 2 (SARS-CoV-2) infection that causes coronavirus disease 2019 (COVID-19) was first declared as pandemic by the World Health Organization in March 2020 and has caused more than 6 million deaths worldwide to date [1]. There is no doubt that critical COVID-19, requiring intensive respiratory support and/or intubation, and mortality strongly associate with advanced age, male sex and the presence of comorbidities [2,3,4,5]. These associations have remained robust during the second year of the pandemic, and even in breakthrough COVID-19 in vaccinated patients [6]. Overall, the mortality rate of patients admitted in hospitals worldwide with COVID-19 has been approximately 17% since the onset of the pandemic [7].

Multicenter studies provide data on large number of patients, which enable the adjustment for multiple risk factors and can inform health policies regarding the general population. However, (a) the inclusion of mixed populations including previously healthy individuals, individuals with multiple comorbidities, vaccinated and unvaccinated individuals, (b) the differences in local pandemic conditions such as access to healthcare services and/or intensive care units, and (c) specific drug administration protocols and/or availability, all make it impossible to have an objective estimate of COVID-19 mortality rates per se.

During the first months of 2020, medical practice and drug administration varied significantly among countries. Following the results of randomized controlled trials, anticoagulation [8], dexamethasone [9] and remdesivir [10] were included in international recommendations and guidelines for the treatment of COVID-19. Moreover, vaccination against COVID-19 which started by the end of 2020 changed dramatically the prognosis of disease limiting hospitalizations, admission to intensive care units and deaths worldwide [11,12,13].

Since September 2020 all patients admitted in our hospital have been uniformly treated according to national recommendations [14]. Herein, in a single-center study, firstly, we searched for laboratory predictors of critical COVID-19 in a derivation cohort of selected unvaccinated patients without comorbidities in order to avoid associated biases. After creating a prognostic score for critical COVID-19 development in this selected cohort, we examined how this score performs in two cohorts, comprising unvaccinated consecutive patients predominantly infected with the alpha SARS-CoV-2 variant, and consecutive patients predominantly infected with the delta SARS-CoV-2 variant, irrespective of vaccination status.

## 2. Methods

### 2.1. Study Population

This is a retrospective analysis of prospectively collected data from 1755 consecutive patients with COVID-19 admitted in a single tertiary academic hospital in Athens, Greece between 16 September 2020 and 20 December 2021, covering the second, third (alpha variant) and fourth (delta variant) pandemic waves. All patients admitted to our Hospital had findings of pneumonia on chest imaging and/or reduced oxygen saturation (<94%).

#### 2.1.1. Derivation Cohort: Unvaccinated Population without Comorbidities

Two-hundred-forty-one (241) consecutive unvaccinated patients presenting at Laiko University Hospital, Athens, Greece with COVID-19 between September 2020 and September 2021 and free history of any comorbidity or drug use, were included in this study. Comorbidities included known cardiovascular risk factors (arterial hypertension, dyslipidemia), diabetes, physician-assessed obesity, cancer, systemic immune-mediated disease, chronic obstructive pulmonary disease (COPD), asthma or any other lung disease, thyroid disease, kidney or liver disease or the systematic use of any medication including antidepressants and/or anxiolytics.

Demographics of the derivation cohort (*n* = 241, 71% men, 49 years median age), clinical condition at admission, time from symptom-onset at admission, as well as baseline values of hemoglobin, white blood cell (WBC) count, neutrophil/lymphocyte ratio, platelet count, C-reactive protein (CRP), ferritin, fibrinogen, lactate dehydrogenase (LDH), d-dimers, aspartate aminotransferase (SGOT/AST), alanine aminotransferase (SGPT/ALT), gamma-glutamyl transferase(γ-GT) and creatinine were recorded (Table 1).

All patients were uniformly treated according to national guidelines during the study period [14]. In detail, patients with SpO2 < 94% on room air received dexamethasone (6 mg/day for 10 days or until discharge from hospital if earlier) and remdesivir (200 mg i.v. on day 1 and 100 mg i.v. on days 2–5), while almost all patients received low molecular weight heparin (LMWH) at prophylactic dose (98%; Table 1). The primary endpoint of our study was the proportion of patients who developed critical COVID-19 defined as the need of high-flow nasal oxygen therapy and/or mechanical ventilation (invasive and non-invasive with Bi-level Positive Airway Pressure).

#### 2.1.2. Validation Cohorts

Two validation cohorts were used. The first comprised of 1228 unvaccinated consecutive patients predominantly infected with the alpha SARS-CoV-2 variant who were admitted at Laiko University Hospital, Athens, Greece between September 2020 and June 2021 for COVID-19 (second and third pandemic waves). The second cohort included 527 consecutive patients irrespective of vaccination status, who were admitted between July 2021 and December 2021 (fourth pandemic wave, predominance of the delta SARS-CoV-2 variant) (Supplementary Figure S1). All patients were uniformly treated according to national guidelines during the study period [14]. Critical disease in the validation cohorts was defined as respiratory failure requiring high-flow oxygen/mechanical ventilation and/or systemic inflammatory response syndrome development (sepsis-1 criteria [15]), according to NIH definitions [16].

## 3. Statistical Analysis

Normality of variables was assessed by Kolmogorov-Smirnov and Shapiro-Wilk tests. Continuous variables are presented as mean ± S.D. or median (interquartile range; IQR) when non-normally distributed. Categorical variables are presented as absolute count (valid percentage). Differences between 2 groups were assessed by independent samples *t*-test or the non-parametric Mann Whitney U test for continuous variables, and Fisher’s exact test for categorical variables.

To explore the association of clinical and laboratory features with disease course we used binary logistic regression with disease severity (critical vs. non-critical disease course) as dependent variable and each disease feature as independent variable. Associations are presented as odds ratios (OR) with their corresponding 95% confidence intervals (95% CI). To test for collinearity within the final multivariable model, we calculated the variance inflation factor (VIF) of each independent variable.

Furthermore, we employed receiver operator characteristic (ROC) curves analysis to evaluate the predictive ability of pre-specified exposure variables for discriminating patients with critical vs. non-critical COVID-19. We calculated the area under the curve (AUC) and 95% CI using the trapezoidal rule.

Finally, we set to generate an integer-based scoring system from the coefficients of each independent exposure variable in the fitted multivariable model. In specific, we divided each covariate’s coefficient with the smallest coefficient and then rounded to the nearest integer; the score was calculated as the sum of the covariates’ weighted scores. To validate the score, we examined calibration (i.e., the agreement between predicted and observed outcome) using the Hosmer-Lemeshow goodness-of-fit test with 10 groups and discrimination between patients with and without critical COVID-19 by calculating AUC. To increase robustness of our findings, we performed internal validation of the discriminative ability of the simple score: after random splitting of the database, we fitted the multivariable model in the training set (*n* = 120) and calculated the score as previously described. Then, we applied the score in the test set (*n* = 121) and calculated AUC for predicting critical COVID-19. We repeated this loop 500 times and pooled all results to yield the cross-validated AUC.

Statistical analysis was conducted with Stata v. 13, SPSS v. 27 and GraphPad Prism v. 7.05. All tests were 2-tailed. We deemed statistical significance at *p* < 0.05. The study was approved by the Ethics Committee of Laiko University Hospital, Athens, Greece (Protocol Number: 765/2021).

## 4. Results

### 4.1. Laboratory Features on Admission Associated with Critical COVID-19 in the Absence of Comorbidities

A total of 241 patients with no comorbidities nor risk factors for severe disease [71% men, age range: 18 to 82 years, median (IQR): 49 (17) years] were included in the study. Clinical and laboratory features of individuals who had SpO2 > 94% and required no corticosteroids or patients with hypoxia (SpO2 < 94%) who did not require intensive respiratory support (non-critical; *n* = 219) vs. patients who required high-flow oxygen and/or mechanical ventilation (critical disease; *n* = 22) are summarized in Table 1. As shown in Table 1 there were several differences at baseline between patients with non-critical illness and those patients who developed critical illness. While almost all patients had received low molecular weight heparin, dexamethasone and remdesivir were administered in 75/79% of patients with non-critical disease, respectively, as well as in all patients with critical disease (both *p* ≤ 0.01). Tocilizumab was given in 3% of non-critical patients but in 64% of patients with critical disease (*p* < 0.001).

Increased age (OR (95% CI): 1.06 (1.02–1.10) per 1-year increase, *p* = 0.003)), but not male sex, was associated with increased risk of developing critical disease (Table 2). Similarly, increased number of white blood cells and neutrophils (OR (95% CI): 1.13 (1.02–1.24), *p* = 0.018 and 1.26 (1.11–1.43), *p* < 0.001, per 1000 cells/μL increase, respectively), as well as higher neutrophil/lymphocyte ratio [OR (95% CI): 1.21 (1.10–1.34), *p* < 0.001] were also associated with increased risk of developing critical disease (Table 2). Markers of acute inflammatory response including CRP (OR (95% CI): 1.014 (1.008–1.020), *p* < 0.001 per 1 mg/L increase) and ferritin (OR (95% CI): 1.001 (1.000–1.001, *p* = 0.030 per 1 ng/mL increase), hypercoagulability (fibrinogen OR (95% CI): 1.009 (1.005–1.014), *p* < 0.001 per 1 mg/dL increase), cell death (LDH OR (95% CI): 1.007 (1.004–1.010), *p* < 0.001 per 1 U/L increase) or liver dysfunction (γ-GT OR (95% CI): 1.010 (1.004–1.016), *p* = 0.001 per 1 U/L increase) were all associated with increased risk of developing critical disease (Table 2). After adjustment for age, sex and days from symptom-onset, only ferritin lost its association with critical disease development (Table 3).

### 4.2. Predictors of Critical COVID-19 in the Absence of Comorbidities

**Figure 1:**
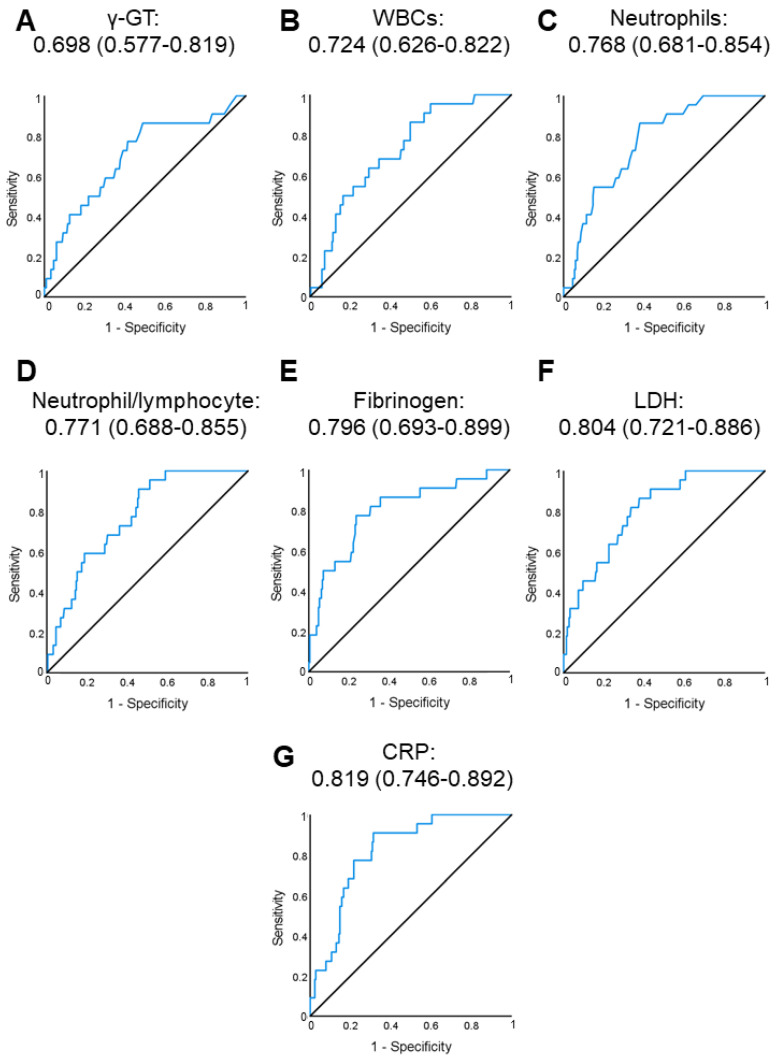
Receiver operating characteristic (ROC) analysis of gamma-glutamyl transferase (γ-GT; (**A**)), white blood cells (**B**), neutrophils (**C**), neutrophil-to-lymphocyte ratio (**D**), fibrinogen (**E**), lactate dehydrogenase (LDH; (**F**)) and C-reactive protein (CRP; (**G**)) for discriminating patients with critical vs. non-critical COVID-19 in the absence of comorbidities (derivation cohort). Numbers represent AUC (95% CI) for each parameter. Abbreviations: AUC: area under the curve, CI: confidence interval.

Next, we used ROC curve analysis to evaluate the predictive ability of each parameter that associated with critical COVID-19 independent of age, sex and time from symptom onset (Table 3), for the discrimination of patients with critical vs. non-critical COVID-19 (Figure 1). All examined parameters showed good predictive ability: γ-GT (AUC (95% CI): 0.698 (0.577–0.819), *p* = 0.002; Figure 1A), WBC count (AUC (95% CI): 0.724 (0.626–0.822), *p* = 0.001; Figure 1B), neutrophil count (AUC (95% CI): 0.768 (0.681–0.854), *p* < 0.001; Figure 1C), neutrophil-to-lymphocyte ratio (AUC (95% CI): 0.771 (0.688–0.855), *p* < 0.001; Figure 1D), fibrinogen (AUC (95% CI): 0.796 (0.693–0.899), *p* < 0.001; Figure 1E), LDH (AUC (95% CI): 0.804 (0.721–0.886), *p* < 0.001; Figure 1F) and CRP (AUC (95% CI): 0.819 (0.746–0.892), *p* < 0.001; Figure 1G).

### 4.3. Development and Validation of a Simple Score for Critical COVID-19 Prediction

As shown in Figure 1, CRP, LDH and fibrinogen were the three parameters with the highest AUC for prediction of critical vs. non-critical COVID-19. We then defined cutoff points with good sensitivity and specificity for the prediction of critical COVID-19 development (Supplementary Table S1). Of note, high CRP (above ROC-defined cutoff), LDH and fibrinogen were independently associated with critical COVID-19 when entered in a multivariable analysis (Supplementary Table S2). Based on the coefficient of each parameter when all three variables were inserted in a multivariable regression model, we next created a score comprising of these 3 standard laboratory biomarkers as follows: Score = 2 × CRP(ROC) + 1 × LDH (ROC) + 1 × fibrinogen(ROC) (Supplementary Table S2).

**Figure 2:**
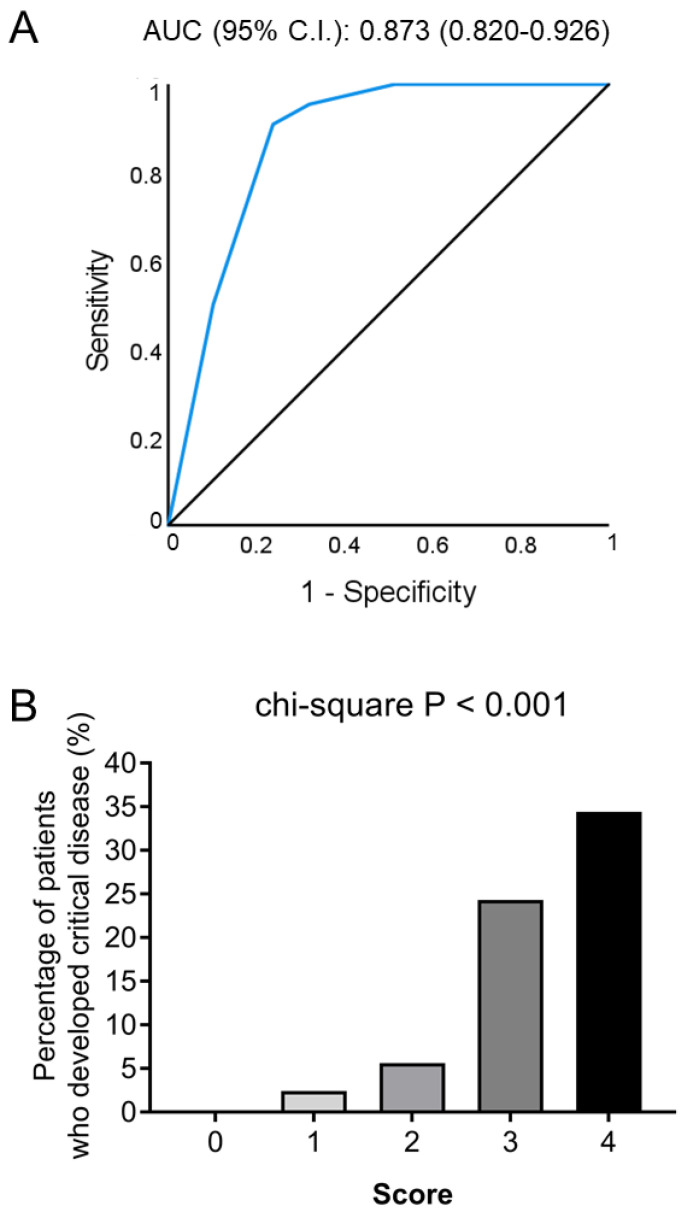
Predictive value of the simple score for the development of critical COVID-19 in the absence of comorbidities (derivation cohort). (**A**). Receiver operating characteristic (ROC) analysis of the simple score for discriminating patients with critical vs. non-critical COVID-19. (**B**). Bars represent the percentage of patients who developed critical COVID-19 according to their score (0–4). Abbreviations: AUC: area under the curve, CI: confidence interval.

We observed that this simple score had a good calibration (Hosmer-Lemeshow test *p* = 0.504) and very good discriminative ability for the detection of patients with critical COVID-19 [AUC (95% CI): 0.873 (0.820–0.926), *p* < 0.001; Figure 2A)]. The score offered a good risk stratification of patients, i.e., one out of three patients with a score of 4 developed critical disease, but none of the patients with a score of 0 did (Figure 2B). By internal validation, this simple score retained its discrimination value for critical COVID-19 (cross-validated AUC: 0.828, 95% CI: 0.745–0.888).

Finally, we examined whether this simple score performed well in 2 unselected cohorts: a first cohort comprising of unvaccinated consecutive patients predominantly infected with the alpha SARS-CoV-2 variant, and a second cohort comprising of consecutive patients predominantly infected with the delta SARS-CoV-2 variant, irrespective of vaccination status. The first validation cohort consisted of 56.5% men, aged 63 (24) (median (IQR)) years. The patients most commonly presented with fever (77.4%), cough (32.8%), dyspnea (29.9%), GI involvement (12.2%) and fatigue (11.3%). Almost all patients received LMWH (97.3%), while 76% and 82% received remdesivir and corticosteroids, respectively. One out of 4 patients (24.4%) developed critical disease, 11.2% received high-flow nasal oxygen supplementation and 8% were intubated, while 1.8% developed pulmonary embolism. The second validation cohort had also male predominance (57.9%) and a similar median age of 63 years (IQR: 26). Again, patients most commonly presented with fever (70.8%), cough (30.2%), dyspnea (27.3%), fatigue (14.2%) and GI involvement (13.3%), and almost all received LMWH (96.4%), while 71% and 75% received remdesivir and corticosteroids, respectively. As also observed in the first validation cohort one out of 4 patients (25.9%) developed critical disease, 10.6% required high-flow oxygen, 8% were intubated, whereas pulmonary embolism was observed in 2.1% of the patients.

**Figure 3:**
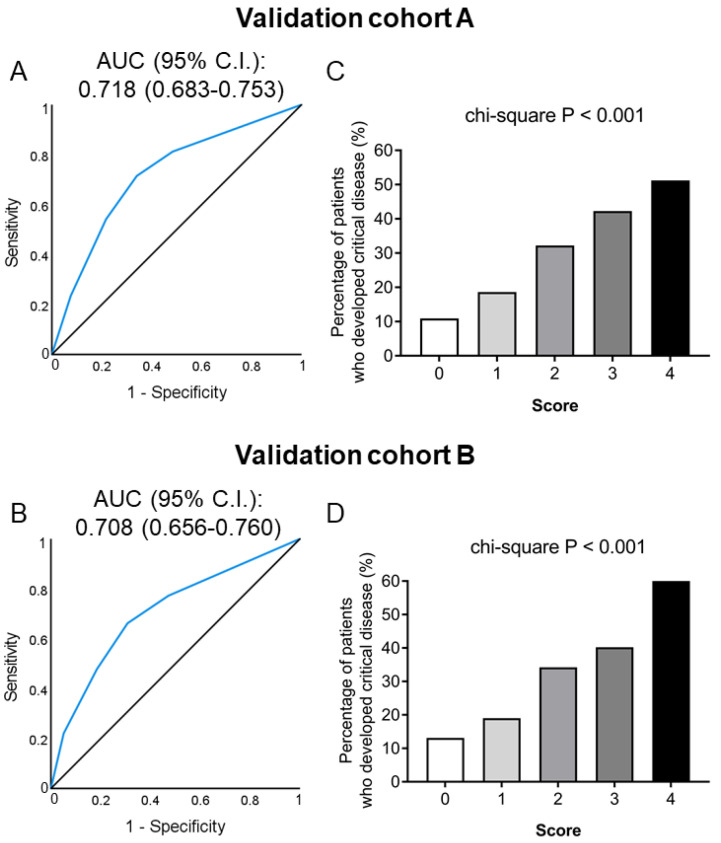
Predictive value of the simple score for the development of critical COVID-19 in a cohort of 1228 unvaccinated patients predominantly infected by the alpha SARS-CoV-2 variant (**A**,**C**) and in a cohort of 527 patients (65% unvaccinated) infected predominantly by the delta SARS-CoV-2 variant (**B**,**D**). Receiver operating characteristic (ROC) analysis of the simple score for discriminating patients with critical vs. non-critical COVID-19 (**A**,**B**). Bars represent the percentage of patients who developed critical COVID-19 (**C**,**D**) according to their score (0–4). Abbreviations: AUC: area under the curve, CI: confidence interval.

As shown in Figure 3, this score performed well in the cohort of 1228 consecutive unvaccinated patients infected by the alpha variant (AUC: 0.718, 95% CI: 0.683–0.753, *p* < 0.001; Figure 3A), as well as in the mixed cohort (*n* = 527, 65% unvaccinated) infected by the delta variant (AUC: 0.708, 95% CI: 0.656–0.760, *p* < 0.001; Figure 3B); only approximately 10% of patients with a score of 0 developed critical disease in contrast with 50–60% of patients with a score of 4 in both cohorts (Figure 3C,D).

## 5. Discussion

In this study we analyzed the prognostic value of routinely-measured laboratory features at admission for the development of critical COVID-19 in a derivation cohort of unvaccinated patients without existing comorbidities who were treated according to protocols standardized after the summer of 2020 [14]. Among 241 hospitalized unvaccinated patients with COVID-19, 22 (9.1%) developed critical disease requiring intensive respiratory support and only 2 died (0.8%). In sharp contrast, mortality of the unselected validation cohorts comprising consecutive patients hospitalized for COVID-19 was 15.6% and 18.1% for the alpha and delta variant, respectively.

A combination of laboratory parameters indicative of acute inflammation (CRP), cell death (LDH), and hypercoagulable state (fibrinogen), showed good discrimination value for the development of critical COVID-19 in the derivation cohort, which was higher than any of the parameters alone. Our study is one of the very few available studies focusing on patients without pre-existing comorbidities [17,18,19,20,21,22] and, to the best of our knowledge, the first one that included patients who were uniformly treated with anticoagulants and corticosteroids/remdesivir in cases with oxygen desaturation [14].

Furthermore, we show that the simple score consisting of CRP, LDH and fibrinogen has an excellent discriminative value for patients with critical vs. non-critical COVID-19, not only in the derivation cohort, but also in two validation cohorts regardless of the predominant SARS-CoV-2 variant (alpha or delta) or vaccination status. Given that this score was developed in patients with absence of comorbidities, we hypothesize that it may reflect the disease course of COVID-19 per se.

Previous large multicenter studies have established the critical role of existing comorbidities for the development of severe disease or death among hospitalized patients who received, or not, variable treatments for COVID-19 [2,5]. Aging, male sex, cardiovascular risk factors and cardiovascular disease, kidney and lung dysfunction, cancer and autoimmune diseases have been associated with worse clinical outcomes among patients with COVID-19 [2,5,23]. In the presence of comorbidities, the classic pneumonia scores such as CURB-65 and PSI [24,25] as well as a quicker clinical score, namely qSOFA [26,27], have shown a good correlation with poor prognosis. However, so far no study has investigated a cohort free of comorbidities and stable admission criteria, homogeneous and stable therapeutic approach. Studying a previously healthy population allows us to examine the effect of SARS-CoV-2 infection per se on laboratory markers, which otherwise can be largely affected by existing comorbidities. As expected, we found that increased age was associated with increased risk of critical disease, which however developed in less than 10% of these patients without underlying medical conditions. Notably, critical disease was not associated with male sex in our selected cohort. On the other hand, the number of female patients was half than men across all age groups, even though in general women have higher probabilities than men to be comorbidity-free. Taken together, these findings suggest that female sex per se may be protective for COVID-19 requiring hospitalization, which is consistent with the notion that women handle viral illnesses better than men [28] and (b) male sex per se may not be associated with development of critical disease in the absence of comorbidities.

In the derivation cohort, a strong innate immune response as characterized by increased neutrophils and neutrophil-to-lymphocyte ratio, as well as increased CRP levels, was associated with critical disease development independently of age, sex and days from symptom onset. In line with our findings, CRP stood out as the single factor associated with severe COVID-19 in multivariable analysis in a nested case-control study of 134 COVID-19 patients [17]. Similarly, increased neutrophils and decreased lymphocytes, and an increased neutrophil-to-lymphocyte ratio have been associated with development of severe disease in the absence of comorbidities [17,19].

Moreover, we show that a hypercoagulable state, as expressed by increased fibrinogen levels, is associated with critical disease development. Previous studies have also shown that hypercoagulability and thrombosis as expressed by prolonged activated partial thromboplastin time (aPTT) [20] or increased d-dimers [19,20,22] are associated with severe COVID-19 development among patients without comorbidities. A high rate of thromboses is observed among patients with COVID-19, while even patients who have been released from the hospital are in increased risk of thrombosis in the next months [29].

Notably, cell death/tissue damage as expressed by increased serum LDH was also a strong predictor of critical disease development in the derivation cohort. Increased LDH release in the circulation could reflect either direct infection of cells/tissues by SARS-CoV-2 or extensive tissue damage secondary to a strong systemic inflammatory response. Nevertheless, LDH seems to be an independent predictor for severe disease development and mortality consistently reported in patients without existing comorbidities [17,18,20,22], while it has not been included in COVID-19 risk scores such as the well-established 4C mortality score [2], which was developed based on mixed populations. This discrepancy further underlines the need to study patients without pre-existing comorbidities.

Our study has some limitations. First of all, the size of the derivation cohort of patients without comorbidities was modest and only 22 patients developed a critical course, which is somehow expected, thus, the multivariate analysis is weak. Moreover, only 2 patients died in the derivation cohort, which did not allow us to build a prognostic model for death. Therefore, we only used multivariable analysis to examine the predictive ability of each parameter associated with critical COVID-19 independent of age, sex and time from symptom onset (Table 3), while ensuring a ratio of 5 observations per variable in the model. Moreover, whether our score will remain accurate over multiple variants (e.g., the currently predominant Omicron variant), cohort vaccination status, treatment bias and timeline in the pandemic remains to be determined in future studies.

To conclude, we propose that a combination of standard peripheral blood biomarkers of acute inflammatory response, cell death and hypercoagulability, i.e., CRP, LDH and fibrinogen, reflects the severity of COVID-19 per se independently of comorbidities, age and sex, being of value for risk stratification in unselected patients. Given the routine use of these biomarkers in clinical practice, this prognostic score for critical COVID-19 could help in risk stratification of patients. Its prognostic value in unselected COVID-19 cohorts comprising vaccinated populations, as well as its predictive value for mortality in combination with age, deserves further studies.

## Figures and Tables

**Table 1:** Characteristics of the derivation study cohort at hospital admission, stratified also by the subsequent need of high-flow oxygen and/or intubation (critical disease course).

	Derivation Total Cohort (*n* = 241)	Non-Critical Course (*n* = 219)	Critical Course (*n* = 22)	*p*-Value *
**Demographics**				
Male sex	172 (71.4)	155 (70.8)	17 (77.3)	0.626
Age (years)	49 (17)	48 (17)	57 (20)	0.005
**Days from symptom onset to admission**	7 (4)	7 (5)	7 (4)	0.164
**Symptoms and signs at admission**				
Dyspnea (subjective)	105 (43.6)	90 (41.1)	15 (68.2)	0.022
Cough	104 (43.2)	96 (43.8)	8 (36.4)	0.653
Fever	208 (86.3)	188 (85.8)	20 (90.9)	0.747
Fatigue	30 (12.4)	29 (13.2)	1 (4.5)	0.327
Myalgia	16 (6.6)	15 (6.8)	1 (4.5)	1.000
Headache	21 (8.7)	20 (9.1)	1 (4.5)	0.702
Anosmia/Ageusia	8 (3.3)	8 (3.7)	0 (0)	1.000
SpO2 < 94% at admission	194 (80.5)	172 (78.5)	22 (100)	0.010
**Laboratory parameters at admission**				
Hemoglobin (g/dL)	14.3 (2.1)	14.3 (2.1)	14.4 (1.3)	0.622
WBCs (K/μL)	5.6 (3.4)	5.5 (3.1)	8.3 (4.2)	0.001
Neutrophils (K/μL)	4.1 (3.0)	3.9 (2.7)	6.9 (4.1)	<0.001
Lymphocytes (K/μL)	1.05 (0.68)	1.1 (0.7)	0.93 (0.56)	0.095
Neutrophil/lymphocyte ratio	3.8 (3.3)	3.5 (3.0)	7.2 (6.6)	<0.001
Platelets (K/μL)	192 (98)	190 (93)	218 (89.5)	0.007
D-dimers (μg/mL)	0.72 (0.63)	0.69 (0.61)	0.97 (0.77)	0.011
Fibrinogen (mg/dL)	528 (181)	516 (169)	690 (165)	<0.001
Creatinine (mg/dL)	0.85 (0.25)	0.85 (0.25)	0.87 (0.23)	0.349
AST (U/L)	35 (24)	34 (22)	48 (28)	0.001
ALT (U/L)	29 (28)	28 (25)	46 (51)	0.012
γ-GT (U/L)	33 (47)	31 (42)	62 (112)	0.002
LDH (U/L)	322 (166)	311 (156)	450 (251)	<0.001
CRP (mg/L)	41 (74)	39 (66)	112 (77)	<0.001
Ferritin (ng/mL)	507 (618)	477 (591)	764 (797)	0.014
**Drug treatment**				
LMWH	237 (98.3)	215 (98.2)	22 (100)	1.000
Dexamethasone	194 (80.5)	172 (78.5)	22 (100)	0.010
Remdesivir	185 (77.1)	163 (74.8)	22 (100)	0.003
Tocilizumab	21 (8.7)	7 (3.2)	14 (63.6)	<0.001

Continuous variables are presented as median (interquartile range) and categorical variables as absolute count (valid percentage). * *p*-value refers to the comparison of critical vs. non-critical patients. Continuous variables were compared with the use of Mann Whitey U test and categorical variables with Fisher’s exact test. Abbreviations: WBCs: white blood cells; AST: aspartate aminotransferase; ALT: alanine aminotransferase; γ-GT: gamma-glutamyl transferase; LDH: lactate dehydrogenase; CRP: C-reactive protein; LMWH: low molecular weight heparin.

**Table 2:** Univariable association of age, sex and baseline laboratory parameters with critical COVID-19 in the absence of comorbidities (derivation cohort).

	OR (95% CI) *	*p*-Value
Male sex	1.40 (0.50–3.97)	0.522
**Age (years)**	**1.06 (1.02–1.10)**	**0.003**
Time from symptom onset (days)	1.11 (0.96–1.28)	0.148
Hemoglobin (g/dL)	1.11 (0.82–1.50)	0.488
**WBCs (K/μL)**	**1.13 (1.02–1.24)**	**0.018**
**Neutrophils (K/μL)**	**1.26 (1.11–1.43)**	**<0.001**
Lymphocytes (K/μL)	0.47 (0.17–1.25)	0.128
**Neutrophil/lymphocyte ratio**	**1.21 (1.10–1.34)**	**<0.001**
Platelets (K/μL)	1.004 (1.000–1.008)	0.053
D-dimers (μg/mL)	1.11 (0.97–1.26)	0.123
**Fibrinogen (mg/dL)**	**1.009 (1.005–1.014)**	**<0.001**
Creatinine (mg/dL)	1.57 (0.36–6.79)	0.548
AST (U/L)	1.01 (0.999–1.024)	0.080
ALT (U/L)	1.005 (0.998–1.012)	0.178
**γ-GT (U/L)**	**1.010 (1.004–1.016)**	**0.001**
**LDH (U/L)**	**1.007 (1.004–1.010)**	**<0.001**
**CRP (mg/L)**	**1.014 (1.008–1.020)**	**<0.001**
**Ferritin (ng/mL)**	**1.001 (1.000–1.001)**	**0.030**

* Odds ratios are derived from binary logistic regression with critical COVID-19 as the dependent variable, and per one-unit change in the variable depicted in each row as the independent variable. Abbreviations: OR: odds ratio; CI: confidence interval; WBCs: white blood cells; AST: aspartate aminotransferase; ALT: alanine aminotransferase; γ-GT: gamma-glutamyl transferase; LDH: lactate dehydrogenase; CRP: C-reactive protein.

**Table 3:** Association of laboratory parameters at hospital admission with critical COVID-19 after adjustment for age, sex and time from symptom onset in the absence of comorbidities (derivation cohort).

	OR (95% CI) *	*p*-Value
WBCs (K/μL)	1.13 (1.02–1.25)	0.019
Neutrophils (K/μL)	1.23 (1.08–1.41)	0.002
Neutrophil/lymphocyte ratio	1.20 (1.08–1.33)	0.001
Fibrinogen (mg/dL)	1.008 (1.004–1.013)	<0.001
γ-GT (U/L)	1.010 (1.003–1.016)	0.004
LDH (U/L)	1.007 (1.004–1.010)	<0.001
CRP (mg/L)	1.012 (1.006–1.019)	<0.001
Ferritin (ng/mL)	1.000 (0.9999–1.001)	0.129

* Odds ratios are derived from multivariable logistic regression with critical COVID-19 as the dependent variable, and per one-unit change in the variable depicted in each row plus age, sex and time from symptom onset (days) as independent variables. Abbreviations: OR: odds ratio; CI: confidence interval; WBCs: white blood cells; γ-GT: gamma-glutamyl transferase; LDH: lactate dehydrogenase; CRP: C-reactive protein.

## Supplementary Materials

The following supporting information are available online at https://www.mdpi.com/article/10.3390/jcm11071810/s1, Table S1. Sensitivity and specificity of ROC-defined cutoffs for fibrinogen, LDH and CRP used to calculate the score for prediction of critical disease in the absence of comorbidities (derivation cohort), Table S2. Association of high CRP, LDH and fibrinogen (above ROC-defined cutoffs) with critical COVID-19 in the absence of comorbidities (derivation cohort). Figure S1. Flow-chart of the study design.
