# Supplementary material for: A Simple Prognostic Score for Critical COVID-19 Derived from Patients without Comorbidities Performs Well in Unselected Patients
Source: J Clin Med. 2022 Mar 25;11(7):1810. doi: 10.3390/jcm11071810 (PMC8999885; doi:10.3390/jcm11071810)

Supplementary file for

A simple prognostic score for critical COVID-19 derived from patients without comorbidities performs well in unselected patients

Vasiliki E.Georgakopoulou[1*], Nikolaos I. Vlachogiannis[2*], Dimitrios Basoulis[1], Irene Eliadi[1], Georgios Georgiopoulos[3], Georgios Karamanakos[1], Sotiria Makrodimitri[1], Stamatia Samara[1], Maria Triantafyllou[1], Pantazis M. Voutsinas[1], Fotinie Ntziora[2], Mina Psichogiou[1], Michael Samarkos[1], Petros P. Sfikakis[2], Nikolaos V.Sipsas[1,4]

1. Infectious Diseases and COVID-19 Unit, General Hospital of Athens Laiko, Medical School, National and Kapodistrian University of Athens, 11527 Athens, Greece.

2. First Department of Propaedeutic Internal Medicine and Joint Academic Rheumatology Program, Medical School, National and Kapodistrian University of Athens, 11527 Athens, Greece.

3. Department of Clinical Therapeutics, Medical School, National and Kapodistrian University of Athens, 11528 Athens, Greece.

4. Pathophysiology Department, Medical School, National and Kapodistrian University of Athens, 11527 Athens, Greece.

*equal contribution

Short title: Prognostic score for critical COVID-19

Correspondence: nsipsas@med.uoa.gr

**Table S1. Sensitivity and specificity of ROC-defined cutoffs for fibrinogen, LDH and CRP used to calculate the score for prediction of critical disease in the absence of comorbidities (derivation cohort).**		
	**Sensitivity (%)**	**Specificity (%)**
Fibrinogen > 616.5 mg/dL	77.3	76.4
LDH > 380.5 U/L	77.3	68.3
CRP > 61.765 mg/L	90.9	68.7
Abbreviations: ROC: receiver operator characteristic; LDH: lactate dehydrogenase , CRP: C-reactive protein.		

**Table S2. Association of high CRP, LDH and fibrinogen (above ROC-defined cutoffs) with critical COVID-19 in the absence of comorbidities (derivation cohort).**			
	**Coefficient***	**OR (95% CI)***	**P-value**
Fibrinogen > 616.5 mg/dL	1.33	3.79 (1.17-12.29)	0.026
LDH > 380.5 U/L	1.24	3.47 (1.12-10.71)	0.031
CRP > 61.765 mg/L	2.01	7.43 (1.44-38.34)	0.017
* Coefficients and odds ratios were calculated using binary logistic regression with critical COVID-19 as the dependent variable and higher than ROC-defined cutoff fibrinogen, LDH or CRP as independent variables.<br>Abbreviations: OR: odds ratio, CI: confidence interval, LDH: lactate dehydrogenase , CRP: C-reactive protein.			

Figure S1. Flow-chart of the study design.

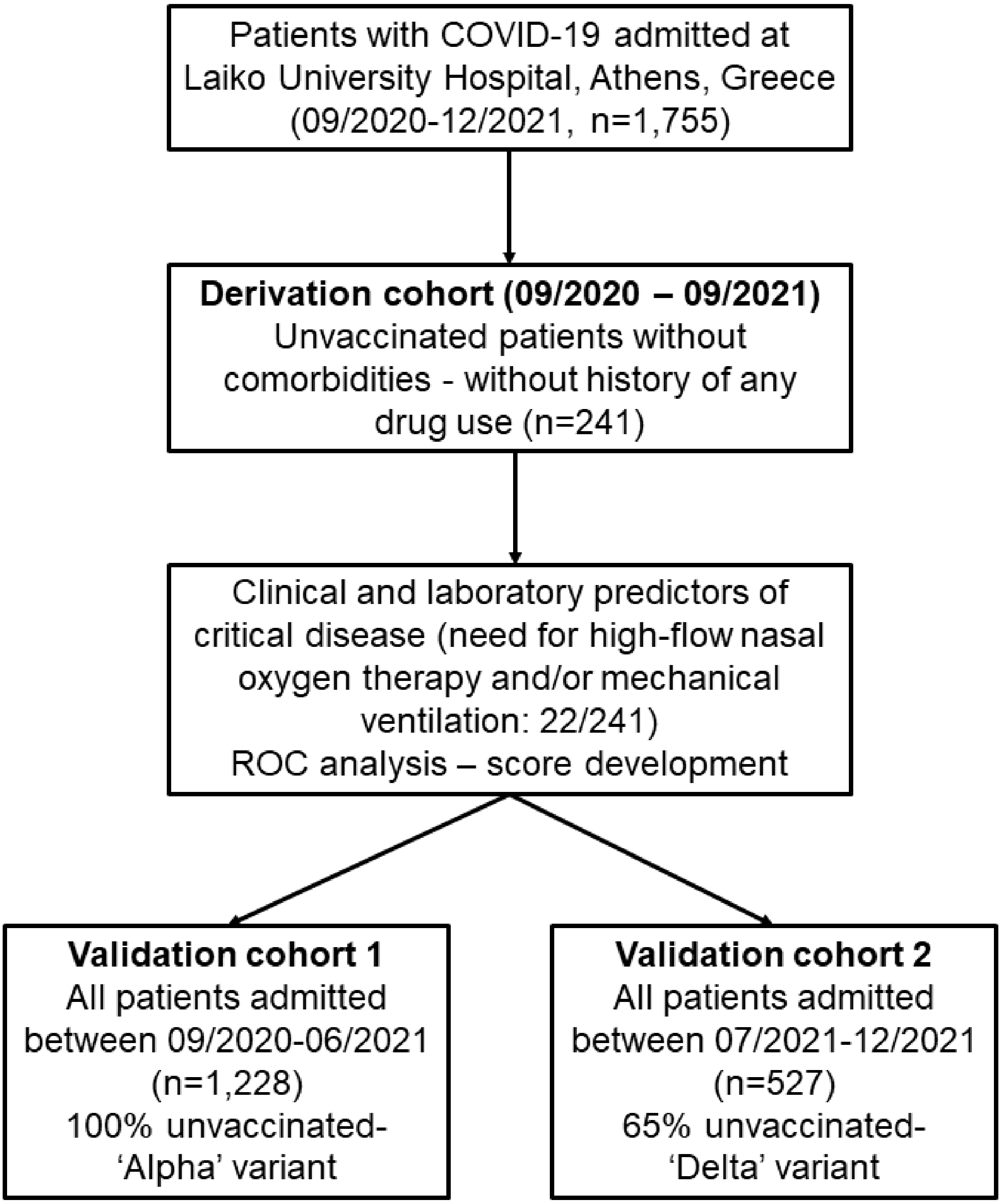

Supplement: Supplementary file 1 [file jcm-11-01810-s001.zip › jcm-1602416-supplementary.pdf]
